# *De novo* assembly and functional annotation of Henbit (*Lamium amplexicaule*) transcriptome

**DOI:** 10.3389/fgene.2025.1612607

**Published:** 2025-10-28

**Authors:** Young Ji Choi, Hyojung Son, Jaewon Lim, Siyoon Jeong, Seongmin Oh, Bomi Nam, Kang-Yeol Yu, Kyung Min Choi, Myunghee Jung, Ha Yeun Song

**Affiliations:** ^1^ Advanced Research Center for Island Wildlife Biomaterials, Honam National Institute of Biological Resources, Mokpo-si, Republic of Korea; ^2^ Research and Development Center, Insilicogen Inc., Yongin-si, Republic of Korea

**Keywords:** *de novo* assembly, *Lamium amplexicaule*, transcriptome, secondary metabolism, biotic and abiotic stress

## Introduction

Henbit, scientifically known as *Lamium amplexicaule*, is a winter annual weed from the Lamiaceae family, native to Europe, Asia, and North Africa. This plant holds considerable value in traditional medicine. The Lamiaceae family is frequently cited in ethnobotanical research as one of the most utilized plant families for medicinal purposes, with its potential medicinal properties and traditional uses being extensively studied (Alipieva et al., 2006; Bubueanu et al., 2019; Kachmar et al., 2021). For instance, a survey in Taza, Morocco identified the Lamiaceae family as the most frequently used for traditional medicine (Kachmar et al., 2021). Various Lamium species, particularly *Lamium album* and *Lamium maculatum*, have a long-standing history in folk and traditional medicine across cultures. *L. album* has traditionally been used as a blood tonic, anti-spasmodic, and anti-inflammatory agent (Alipieva et al., 2006). In contrast, *L. maculatum* has been employed in Chinese folk medicine to treat trauma, fracture, and hypertension (Alipieva et al., 2006). Research has explored the haemostatic properties of butanolic extracts from these species, showing potential in blood clotting applications (Bubueanu et al., 2019). The medicinal uses of Lamium species are diverse, with *L. album* and *Lamium purpureum* being used in both human and veterinary traditional medicine, utilizing aerial parts and roots (Bubueanu et al., 2019). This plant contains several bioactive compounds, including flavonol glycosides (Nugroho et al., 2009), and iridoid glucosides such as lamalbid, sesamoside, and lamioside (Adema, 1968; Kobayashi et al., 1986; Alipieva et al., 2003; Alipieva et al., 2007) and phytol, β-sitosterol, isorhamnetin, hydroxynervonic acid, and phenolic components have been isolated from *L. amplexicaule* (Ghoneim et al., 2018; Siham and Rachid, 2022). These compounds contribute to the plant’s biological activities. Notably, *L. amplexicaule* has demonstrated promising antimicrobial properties, especially against methicillin-resistant *Staphylococcus aureus* (MRSA) (Ghoneim et al., 2018). Compounds such as phytol, isorhamnetin, and 3,4-dihydroxy-methyl benzoate extracted from the plant showed significant anti-MRSA effects (Ghoneim et al., 2018). Additionally, the mechanism of action against the dehydro-squalene synthase enzyme was established, suggesting potential for developing new anti-MRSA candidates.

In terms of plant agronomy, although *L. amplexicaule* is often regarded as a weed, it has attracted scientific interest due to its invasive nature, unique reproductive strategies, and role as an alternative host for agricultural pests. The species exhibits remarkable pheno-plasticity, particularly in its flower organs, with both cleistogamous (closed) and chasmogamous (open) flowers (Johnson et al., 2008). Researchers found, *L. amplexicaule* plant inhibited root and shoot growth of various species, including *Lepidium sativum* and *Lolium multiflorum*, with methyl caffeate identified as a phytotoxic substance with allelopathic activity (Jones et al., 2012; Sakamoto et al., 2019). Additionally, the *L. amplexicaule* has been identified as a host for the soybean cyst nematode (*Heterodera glycines* Ichinohe, SCN), a significant pest in soybean production (Ramarao Venkatesh et al., 2000; Johnson, et al., 2008). Furthermore, its attractive flowers, which draw pollinators and birds, combined with its ability to thrive in diverse climates, have made it a popular choice for landscaping, vegetation restoration, and ornamental gardening purposes (Binder et al., 2024; Stojanova et al., 2024; Zhou et al., 2024). Furthermore, plant-derived extracts containing high levels of phytotoxic compounds, such as methyl caffeate, were observed to suppress the growth of roots and shoots in various plant species, contributing to the allelopathic effect (Sakamoto et al., 2019).

The stated objectives underscore the benefits and importance of cultivating/killing this plant for agricultural purposes and manufacturing nutraceutical products for industrial use. However, research into the genetic components, including genomic and transcriptomic aspects, remains scarce within this plant family. The scarcity of sequencing libraries in the NCBI public genetic database results in a dearth of published information for comprehending gene composition and identifying secondary metabolism-related genes in these plants. In the current genomics era, elucidating genetic elements for plants lacking a reference genome through *de novo* transcriptome assembly could offer a cost-efficient method to acquire preliminary data for any plant species. This research seeks to bridge the knowledge gap in genetic elements of the plant family by employing *de novo* transcriptome assembly techniques. Through the generation of a transcriptome, the scientists aim to reveal crucial information about secondary metabolism transcripts, potentially shedding light on the plant’s applications in agriculture and industry. This strategy not only provides an economical solution for examining plants without a reference genome but also lays the groundwork for future studies on gene composition and secondary metabolism-related genes within the Lamiaceae family.

## Materials and methods

### Stressed plant materials and RNA-sequencing

**FIGURE 1:**
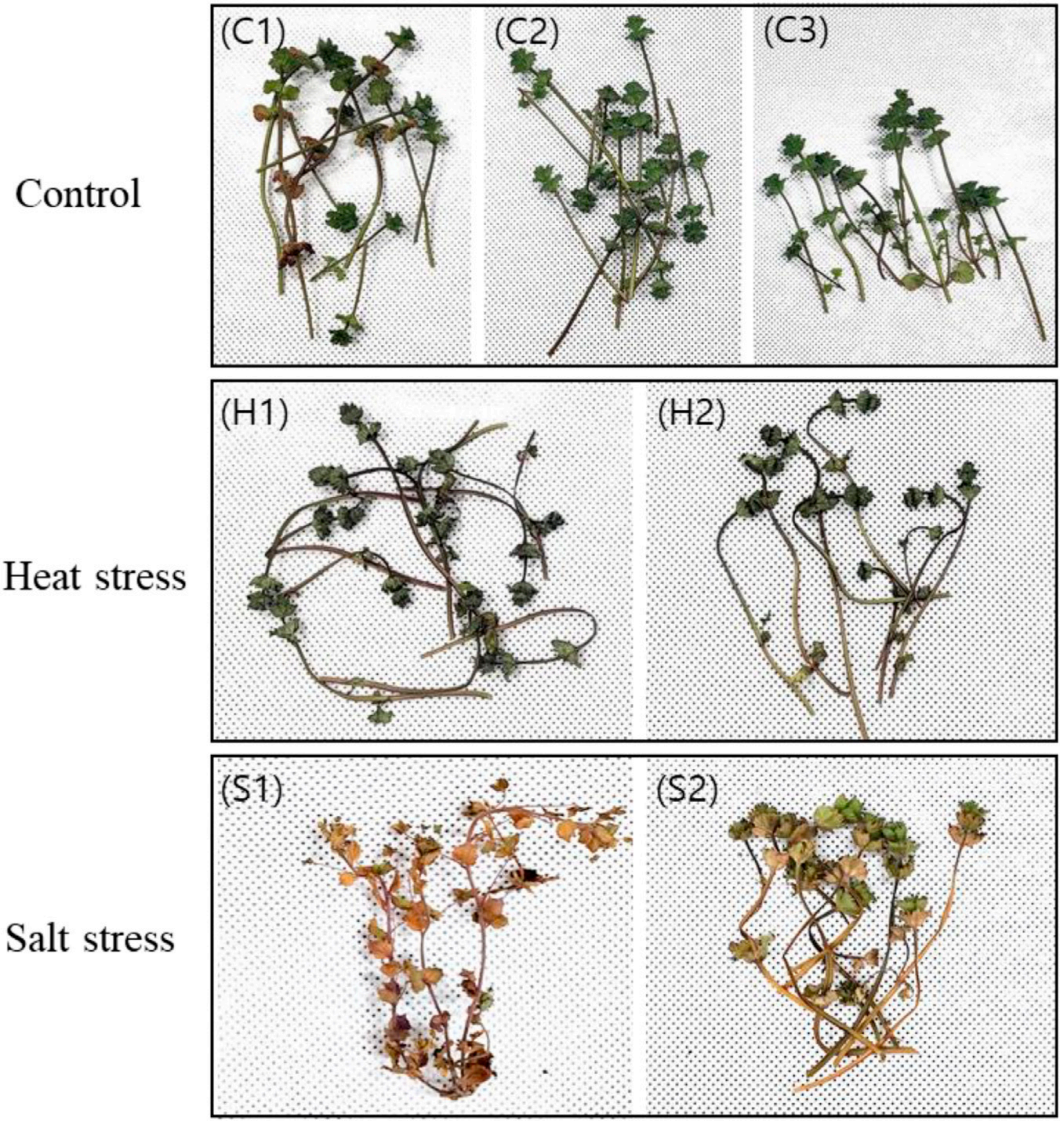
Phenotypic alterations in *Lamium amplexicaule* (LA) under conditions of heat and salt stress. The control group (C1, C2, C3) did not undergo any treatment. Heat stress was induced at 35 °C for a duration of 3 days (H1, H2), while salt stress was applied using seawater with a salinity of 34‰ for 14 days (S1, S2). C: control; H: heat; S: salt.

In March 2023, *L. amplexicaule* was collected from Mokpo, Korea (34°76′N, 126°36′E). The plants were acclimated for 2 weeks in 12 cm diameter pots filled with culture soil, maintained at 25 °C ± 2 °C under a 16-h light/8-h dark photoperiod. After acclimation, heat stress treatment was applied by placing 2 *L. amplexicaule* plants in a 35 °C incubator (Multi-room Incubator, VISION) with a 16-h light/8-h dark photoperiod for 3 days, while three plants remained at 25 °C as controls. A separate set of two plants was used for the salt stress treatment, 200 mL of seawater (salinity of 34‰) was applied daily for 14 days, and their physiological responses were monitored throughout the experiment (Figure 1). Post-treatment, leaves were collected for sampling. Control leaf samples were labeled C1, C2, and C3; those subjected to heat stress were labeled H1 and H2; and those subjected to salt stress were labeled S1 and S2 (Figure 1). Fresh samples were immediately frozen in liquid nitrogen and stored at −80 °C for subsequent experimental analyses. Total RNA was extracted from different tissue parts of *L. amplexicaule* using the Trizol method (Lian et al., 2024) and sequenced with the Illumina Next-Seq. The entire process was outsourced to Macrogen, South Korea.

### *De novo* transcriptome assembly, functional annotation and differential expression analysis

The raw data obtained underwent filtration to exclude reads containing more than 5% N-base content, reads with low-quality base counts exceeding 50%, and reads containing adapter contamination and repetitive sequences resulting from PCR amplification. Subsequently, the processed short read sequences were subjected to contig assembly with well optimized transcriptome assembler Trinity and translated with TransDecoder (Haas et al., 2013). Finally, the translated proteins sequences were subjected to homology search, with existing annotation databases (GO, KEGG, Uniprot) were employed to annotate the transcriptome function with Trinotate (Bryant et al., 2017). Further, Differential expression analysis was performed using the read count data of unigene expression from each sample, obtained through expression quantification. Transcript-level quantification was performed using Salmon, and differential expression analysis was conducted using edgeR (Robinson et al., 2010), which employs empirical Bayes methods to estimate gene-wise dispersion and improve statistical reliability, particularly under low-replicate conditions (Chen et al., 2014). Differentially expressed transcripts were filtered using a threshold of adjusted *p-value* (FDR) ≤ 0.05 and |log_2_ fold change| ≥ 2.

### Preliminary analysis report of *L. amplexicaule* transcriptome

**FIGURE 2:**
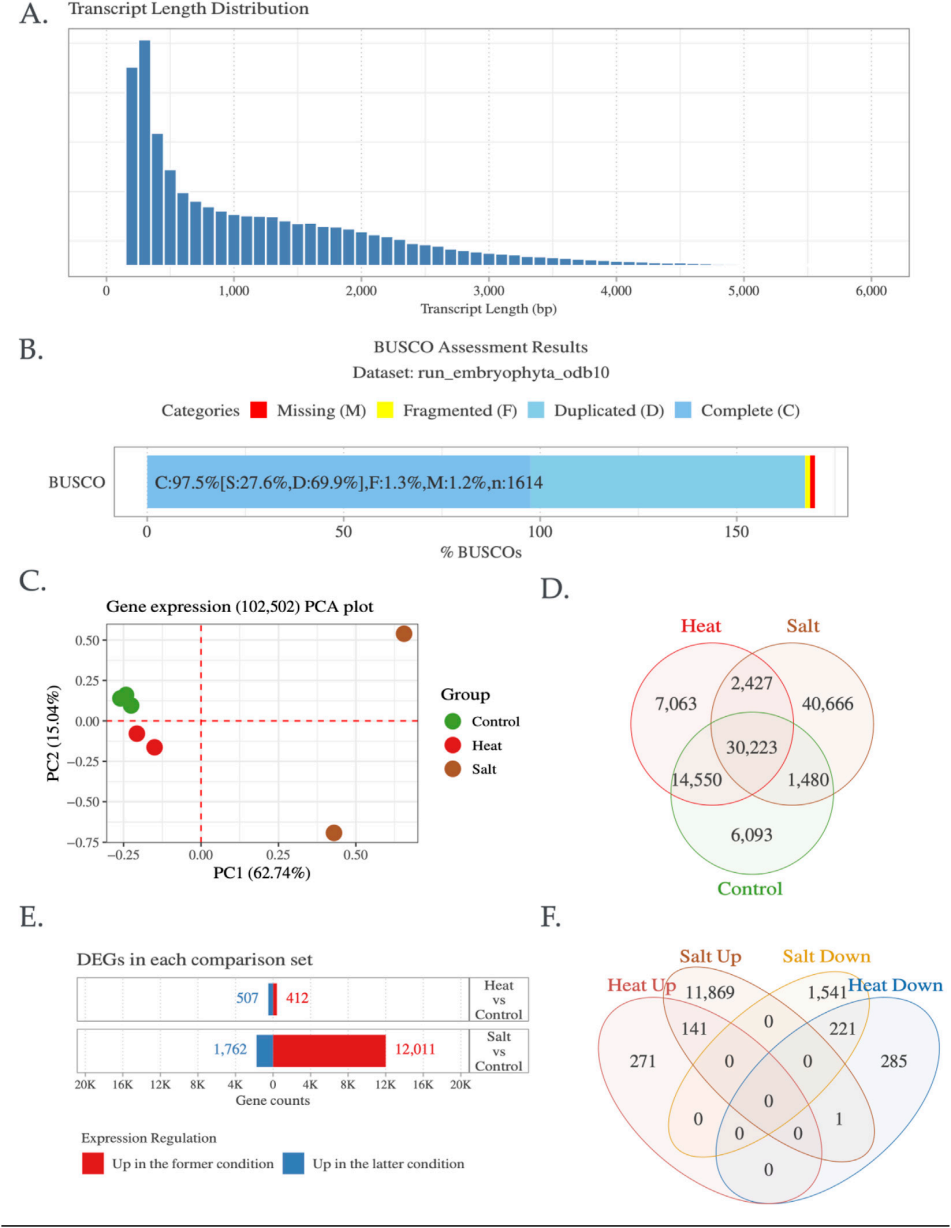
Summary of *Lamium amplexicaule* (LA) *de novo* transcriptome assembly results; **(A)** Transcript length distribution, **(B)** Evaluation of assembly completeness, **(C)** PCA of samples based on expressed 102,502 transcripts, defined as those with read counts ≥10 and TPM ≥0.3 in at least one sample, **(D)** Gene expression changes in response to heat and salt stress, **(E)** Sets of differentially expressed gene sets (log_2_ FC ≥ 1, FDR ≤0.05) under heat and salt stress conditions, and **(F)** Categorization of genes based on specific up- and downregulation patterns.

This study aimed to elucidate the key enzyme genes associated with plant secondary metabolites, adhering to the gene-to-metabolite principle (Osbourn, 2010). RNA was extracted from 7 *L. amplexicaule* leaf samples, and the subsequent cDNA library was sequenced using the Illumina NextSeq high-throughput platform. After filtering, 2 GB of clean reads were obtained. In the absence of *L. amplexicaule* genomic data, Trinity software was utilized for short read assembly and clustering, eliminating redundancy and sequences with ≥95% similarity (Supplementary Figure S1). This process yielded 175,070 transcripts (Table 1), with exhibiting an N50 length of 2,017 bp, lengths spanning 199 to 11,998 bp (Figure 2A), and an average length of 816 bp (Table 1). To ensure the assembled transcriptome completeness the BUSCO (Benchmarking Universal Single-Copy Orthologs) was employed to assess gene completeness (Table 1; Figure 2B), while coding region sequences (CDSs) were predicted for all unique transcripts, resulting in 81,194 complete CDSs (Table 1). To optimize the identification of unique functional genes within the transcriptome, were annotated using multiple databases, including GO, KEGG, and Uniprot (Table 1). Of the 175,070 unigenes, 115,450 (65.9%) were annotated in at least one database, with 116,454 (66.5%) annotated in the GO database and 96,557 (55.2%) in the KEGG database. Further, 102,502 (58.5%) transcripts were expressed across the three experimental groups, including control, heat stress, and salt stress. The expression and differential expression for both stresses were illustrated in Figures 2C–F. Based on the Trinotate annotation and KEGG pathway mapping, 6,595 (5.85%) transcripts were assigned to 28 secondary metabolite pathways (only pathways with more than 10 annotated transcripts were considered), as shown in Supplementary Figure S2. In addition, functional categorization was performed using Mercator4, and the mapping results were visualized with MapMan (Bolger et al., 2021). This analysis focused on secondary metabolism, particularly the triterpenoid biosynthesis pathway, and the corresponding figures are provided as Supplementary Figure S3. Furthermore, to investigate the transcriptional behavior of core gene families involved in secondary metabolism, we focused on cytochrome P450 monooxygenases (PF00067.25) and UDP-glycosyltransferases (UGTs; PF00201.21), which play essential roles in triterpenoid and glycoside biosynthesis. A total of 159 CYP450 and 68 UGT genes were expressed under heat stress, and 166 CYP450 and 71 UGT genes under salt stress. Notably, DEG analysis revealed a stronger transcriptional response under salt stress (Table 2). These families are likely contributing to the biosynthesis of oxygenated and glycosylated triterpenoids, potentially linked to *Lamium*’s antimicrobial and allelopathic properties. Many of these genes mapped to key KEGG pathways, including terpenoid backbone biosynthesis (map00900) and secondary metabolite biosynthesis (map00999), similar to functional modules reported in *Aralia elata* (Cheng et al., 2020). Functional validation may uncover novel genes involved in phytochemical production and stress resilience in *L. amplexicaule*.

**TABLE 1:** Summary of the A: sequencing and assembly; B. Annotations; C: Translation and D. completeness assessment of the transcriptome.

Technology	Reads	Bases
A. Sequencing and assembly		
Raw Sequence	521,929,576	52,714,887,176
Processed Sequence	519,519,220 (99.54%)	52,294,991,673 (99.20%)
*De novo* Assembled Contigs	175,070	218,460,825
Reference Mapped	478,543,262 (92.11%)	48,332,869,462 (91.68%)
Contig N50		2,017
Maximum		19,957
Minimum		201
Total Expressed	102,502 (58.5%)	
Expressed (Control)	52,346 (51.1%)	
Expressed (Heat stress)	54,263 (52.9%)	
Expressed (Salt stress)	74,796 (73.0%)	
B. Annotations		
BLAST Hits	115,450 (65.9%)	
Gene Ontology	116,454 (66.5%)	
KEGG Enzymes	96,557 (55.2%)	
C. Translation		
Total Transcripts	175,070 (100%)	
Complete	81,194 (46.4%)	
5′Partial	32,990 (18.8%)	
3′Partial	14,096 (8.1%)	
Internal	46,790 (26.7%)	
D. BUSCO		
Total Core Genes	1,614 (100%)	
Complete	1,574 (97.5%)	
Fragmented Core Genes	21 (1.3%)	
Missing Core Genes	19 (1.2%)	

**TABLE 2:** Summary of CYP450 (PF00067.25) and UGT (PF00201.21) gene families involved in triterpenoid and glycoside biosynthesis, including KEGG pathway (map00900) associations.

Name	PFAM ID	Condition	Expressed	DEGs	KO00999
CYP450	PF00067.25	Heat stress	159	9	1
CYP450	PF00067.25	Salt stress	166	30	3
UDPGT	PF00201.21	Heat stress	68	1	1
UDPGT	PF00201.21	Salt stress	71	29	8

To further explore secondary metabolites (map00999), we generated a heatmap (Supplementary Figure S4) comprising 181 transcripts, 39 of which showed differential expression under salt or heat stress. Most transcripts were related to the terpenoid pathway, particularly triterpenoid biosynthesis (Kim et al., 2015). As explained in the introduction section, *L. amplexicaule* is known for its glycoside content with therapeutic potential. Many of the identified genes overlap with those found in the ginsenoside biosynthesis pathway, a well-characterized triterpenoid group with demonstrated clinical relevance (Mathiyalagan et al., 2024). Prior studies on Panax ginseng have highlighted the importance of functional group glycosylation (Kim et al., 2015) and enzymes such as dammarenediol synthase (Han et al., 2006) and β-amyrin synthase (Hou et al., 2021), which respond to environmental stresses and drive secondary metabolite biosynthesis. The availability of the complete genome from ginseng facilitates a more comprehensive elucidation of the ginsenoside biosynthesis process. It is well-established that plant scientists predominantly prefer transcriptome datasets for initial research, as advancements in sequencing and sequence assembly methods have been significantly updated to obtain complete transcript lengths and provide detailed insights into the transcripts present in plants. This dataset will facilitate plant scientists’ understanding of the array of genes present in *L. amplexicaule*. The complete expression and differential expression data, along with annotations, were provided in Table 1.

### Value of the data

The significance of the data presented in this transcriptome analysis of *L. amplexicaule* encompasses several aspects: Firstly, it represents the initial comprehensive transcriptome analysis of *L. amplexicaule*, thereby providing valuable genetic information for this medicinally and agriculturally significant plant species. Secondly, it addresses the knowledge gap in genetic elements of the Lamiaceae family, facilitating comparative genomics and evolutionary studies. Additionally, it establishes a foundation for future research on gene functions, particularly those involved in secondary metabolism and antimicrobial properties. Furthermore, it enables targeted genetic improvement and utilization of *L. amplexicaule* for agricultural and industrial purposes. Moreover, it contributes to the understanding of L. amplexicaule’s genetic architecture, which can inform strategies for weed management or cultivation for medicinal purposes. This data is of considerable value to researchers in plant genetics, pharmacology, agriculture, and related fields, as it provides a comprehensive genetic resource for further investigations into this species and its potential applications.

## Limitations

This study has several limitations. First, only two biological replicates were used for each stress condition, limiting statistical power. Second, no qRT-PCR validation was performed to confirm gene expression patterns. Third, functional interpretation was focused mainly on triterpenoid and glycoside pathways. Additionally, while insights from *Panax ginseng* were referenced, they may not fully reflect the biology of *L. amplexicaule*. Lastly, the salt stress treatment (14 days of seawater) may not represent natural field conditions.

## Data Availability

The complete sequences generated in this study have been deposited in the Sequence Read Archive repository under accession number PRJNA1245620 and figshare repository (https://doi.org/10.6084/m9.figshare.28788131), with all annotation details in the Readme file.
