# Supplementary material for: De novo assembly and functional annotation of Henbit (Lamium amplexicaule) transcriptome
Source: Front Genet. 2025 Oct 28;16:1612607. doi: 10.3389/fgene.2025.1612607 (PMC12599991; doi:10.3389/fgene.2025.1612607)
Supplement: Supplementary file 1 [file Presentation1.pptx]

## Slide 1
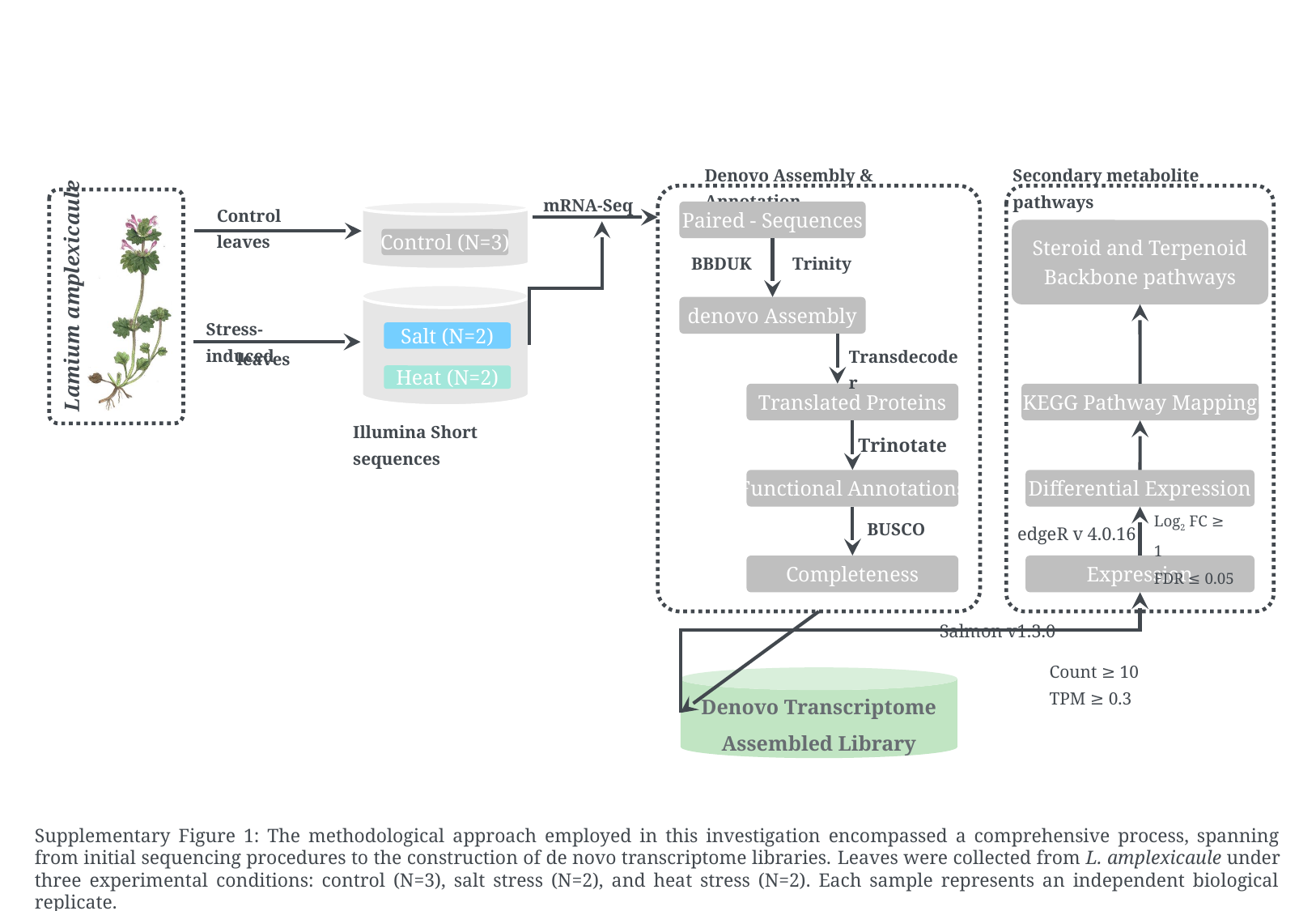

Denovo Assembly & Annotation
Secondary metabolite pathways
mRNA-Seq
Paired - Sequences
Steroid and Terpenoid
Backbone pathways
Control (N=3)
BBDUK
Trinity
Lamium amplexicaule
denovo Assembly
Stress-induced
Salt (N=2)
Transdecoder
leaves
Heat (N=2)
Translated Proteins
KEGG Pathway Mapping
Illumina Short sequences
Trinotate
Functional Annotations
Differential Expression
BUSCO
Expression
Completeness
Denovo Transcriptome
Assembled Library
Control leaves
Log2 FC ≥ 1
FDR ≤ 0.05
edgeR v 4.0.16
Salmon v1.3.0
Count ≥ 10
TPM ≥ 0.3
Supplementary Figure 1: The methodological approach employed in this investigation encompassed a comprehensive process, spanning from initial sequencing procedures to the construction of de novo transcriptome libraries. Leaves were collected from L. amplexicaule under three experimental conditions: control (N=3), salt stress (N=2), and heat stress (N=2). Each sample represents an independent biological replicate.

## Slide 2
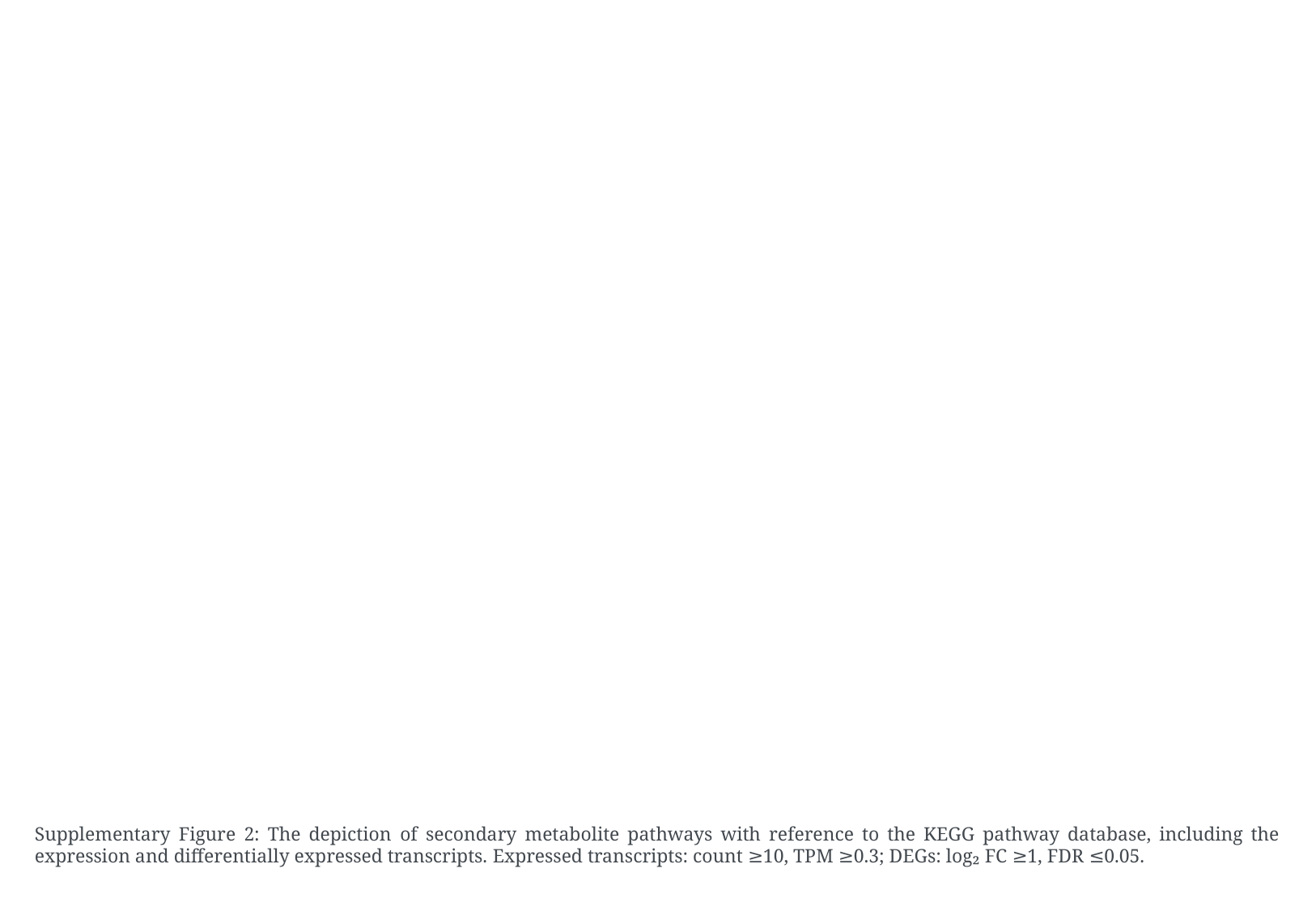

Supplementary Figure 2: The depiction of secondary metabolite pathways with reference to the KEGG pathway database, including the expression and differentially expressed transcripts. Expressed transcripts: count ≥10, TPM ≥0.3; DEGs: log₂ FC ≥1, FDR ≤0.05.

## Slide 3
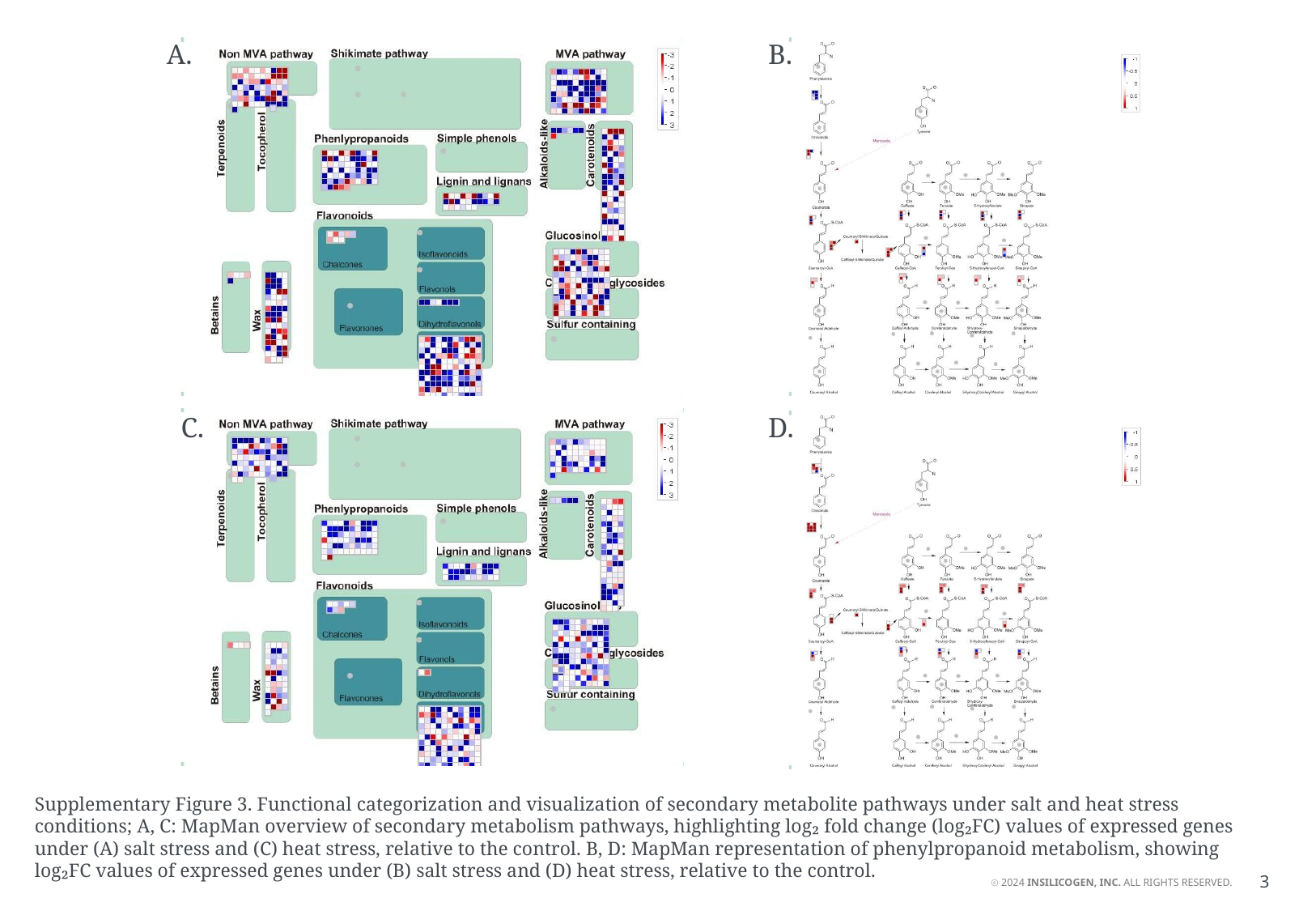

A.
B.
C.
D.
Supplementary Figure 3. Functional categorization and visualization of secondary metabolite pathways under salt and heat stress conditions; A, C: MapMan overview of secondary metabolism pathways, highlighting log₂ fold change (log₂FC) values of expressed genes under (A) salt stress and (C) heat stress, relative to the control. B, D: MapMan representation of phenylpropanoid metabolism, showing log₂FC values of expressed genes under (B) salt stress and (D) heat stress, relative to the control.

## Slide 4
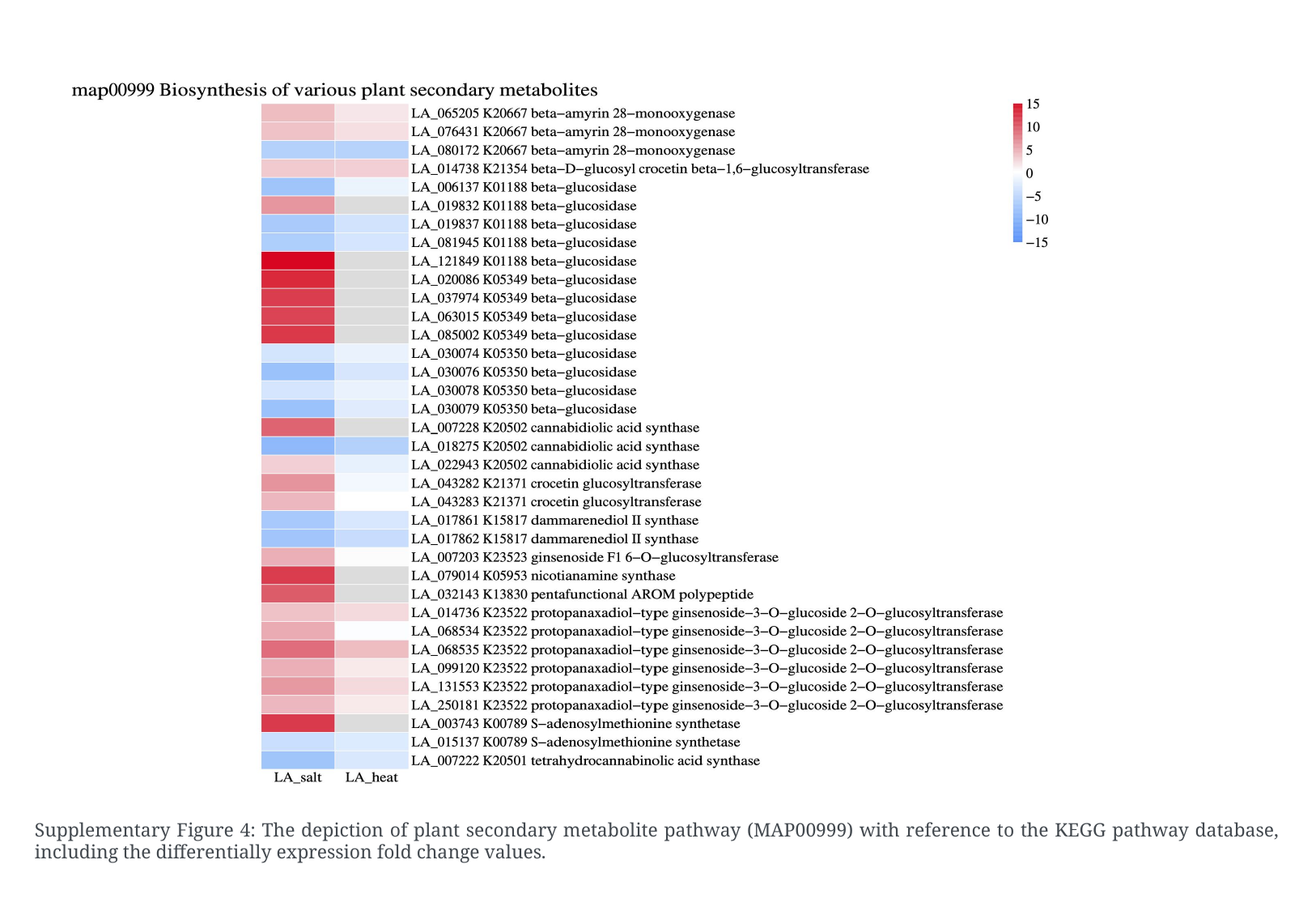

Supplementary Figure 4: The depiction of plant secondary metabolite pathway (MAP00999) with reference to the KEGG pathway database, including the differentially expression fold change values.
